# Power from the press cake: a scoping and compositional review of defatted nut and seed powders with regards to sports nutrition

**DOI:** 10.3389/fnut.2026.1860103

**Published:** 2026-07-08

**Authors:** Charles S. Urwin, Lilia Convit, Zoya Huschtscha, Rhiannon J. Snipe, Vy Tran, D. Lee Hamilton

**Affiliations:** Faculty of Health, School of Exercise and Nutrition Sciences, Institute for Physical Activity and Nutrition, Deakin University, Geelong, VIC, Australia

**Keywords:** anti-nutritional factors, dietary fibre, nut flour, protein quality, sports nutrition, sustainable nutrition

## Abstract

**Purpose:**

Nut and seed press cakes are nutrient-rich by-products of oil extraction, traditionally directed to animal feed. This review synthesises current knowledge on the nutritional composition and functional properties of press cakes, with regards to their incorporation as sustainable protein and fibre rich ingredients in sports nutrition.

**Methods:**

A scoping review was conducted following PRISMA-ScR guidelines to identify studies on nut and seed press cakes relevant to sports nutrition. Nutrient composition and protein quality data were extracted from peer-reviewed sources, with comparisons made between whole nuts/seeds, press cakes, and comparable foods. As only three studies were eligible, the remainder of the review focussed on compositional factors and translation to sports nutrition contexts.

**Results:**

Despite use of press cakes in agricultural contexts for animal feed, and some existing applications in human nutrition, no studies investigated their use for sport, recovery or performance. In comparison to whole nuts/seeds (mean protein content 19.3 g/100 g), protein content was higher in all but one press cake (35.4 g/100 g), with eight press cakes more than doubling the protein content of the whole food equivalent. Energy density was higher in two press cakes; total fat content was lower in all (51.1 g/100 g for whole, 14.3 g/100 g for press cakes). While none exceeded whey protein for leucine or lysine, seven press cakes exceeded WHO leucine guideline. Higher protein and fibre alongside lower energy density and total fat is aligned with some sport nutrition objectives.

**Conclusion:**

Together, there is academic and commercial precedent of press cakes being incorporated into food, especially as baked goods or in smoothies. Their nutritional profiles and compatibility with common foods support their translation to athletes, particularly those following plant-based or environmentally conscious diets, though more evidence is needed to solidify the efficacy of these proposed applications, particularly dose response.

## Introduction

The whey protein global market value is predicted to be $22.63 billion (USD) by 2032, nearly double the 2024 valuation (1). Global valuation figures can be difficult to contextualise, but considering that as recently as the late 20th century whey from cheese production (including the whey protein fractions, lactose and fluid component) was considered a waste product at worst, and cheap animal feed at best (2), this represents an extraordinary shift. Originally a waste product that was highly polluting and costly for dairy processors and cheese makers to dispose of safely (2), whey has since been transformed through technological advances that allow the large-scale separation of its components (proteins, lactose, etc.). Combined with growing recognition of whey protein’s functional and nutritional value, these advances have converted an inconvenient waste into a high value nutrition product. The protein derived from whey alone represents approximately 1% of the global market value of dairy food (1). A key driver of this value is the use of whey protein in sports supplements (1), largely due to its characteristics as a high-quality, rapidly digestible and bioactive protein source (3). Due to their relatively high protein content and minimal processing requirements, there is now growing interest in and market presence of another waste product from the food processing industry: nut and seed press cakes.

Nut and seed press cakes are the solid by-products that remain after oil extraction from whole nuts or seeds, usually through mechanical pressing or solvent-based oil extraction (4, 5). Generally considered waste of the edible oil industry, press cakes can be finely milled to produce a defatted nut/seed flour. This flour can be referred to in several ways depending on source, for almonds and peanuts this defatted flour is often referred to as a “nut butter powder” whereas other manufacturers simply refer to them as defatted nut/seed flours. Regardless of the naming conventions of press cake products, they are increasingly being identified as nutrient-dense materials with potential value beyond animal feed or industrial use (6–8). The nutrient profiles of commonly available nuts and seeds make their press cakes candidates for incorporation into human dietary practices, particularly in contexts where sustainable, high-quality protein and functional food ingredients are relevant (9). They are generally rich in protein, dietary fibre, residual lipids (those remaining after oil extraction such as phospholipids and glycolipids), and bioactive compounds, with composition dependent on source material and processing method (8, 10). These bioactive compounds can include phenolics, lignans, tocopherols, carotenoids and phytosterols (8), contributing to their functional and health-related properties. Extraction methods used to generate nut and seed press cakes influence residual protein composition, typically yielding ~20–50% protein depending on oil removal efficiency and processing conditions. Mechanical, solvent and emerging enzyme/ultrasound-assisted methods differentially impact protein structure and functionality. Readers seeking detailed investigation of these methods are directed to recent reviews on the matter (6, 8).

Nut and seed press cakes are currently primarily directed to animal feed due to their high protein and fibre content (11), providing a cost-effective way to enhance the nutritional quality of livestock diets. In the (human) food industry, they have also been explored as functional ingredients in bakery products, plant-based beverages, snack bars, and protein powders, often serving as a means of modifying texture (12, 13), protein density, or fibre enrichment (8, 9). Beyond food applications, press cakes have found use in biofuel production, composting, and as substrates for fermentation processes to generate enzymes, organic acids, or other products such as biodegradable films (14, 15). Interest has shifted toward upcycling press cakes into human nutrition to reduce waste, with research highlighting their potential as sustainable sources of protein, dietary fibre, and bioactives when incorporated into a food-first approach to nutrition (16).

The accessibility and utilisation of press cakes vary between nut and seed types. Peanut and sunflower seed press cakes are widely used in feed and, to some extent, human food applications due to their large production volumes and developed processing chains (17). Other nut press cakes, such as those from walnuts and almonds, are generated by the nut-oil processing industry (18, 19). Flax, chia, hemp, and pumpkin seed press cakes are more accessible in health food and specialty markets, where they are incorporated into protein-enriched foods and plant-based drinks (20, 21). By contrast, press cakes from macadamia, pecan, and Brazil nut remain niche products, with limited availability likely due to a higher value and cost of the whole nuts. Differences in accessibility and current use are important to consider when evaluating the feasibility of integrating press cakes into human nutrition, as both supply chain consistency and consumer familiarity are likely to contribute to their adoption.

The nutrient composition and amino acid profile ultimately dictate how press cakes can be considered useful within human nutrition, particularly in the context of sports nutrition. High energy and fat content may be advantageous in endurance contexts where caloric density is required (22), while carbohydrate and fibre can influence glycogen availability, satiety and gastrointestinal tolerance (23). With respect to protein, not only is quantity a key consideration, but so too is the digestibility and content of certain amino acids, especially the essential amino acids (EAA). For instance, the leucine content of a protein is important as an EAA and as a key factor in determining the maximal rate muscle protein synthesis after consuming that protein (24, 25). Similarly, lysine, another EAA, is often limiting in plant-based protein sources, and is important for skeletal muscle recovery and repair (26). The other EAA often limiting in plant based sources of protein is methionine which contributes to antioxidant function and metabolism (27). The macronutrient profile and content of specific amino acids could provide the basis for evaluating the utility of nut and seed press cakes in a sports nutrition context.

While general nutrition emphasises health and disease prevention, sports nutrition focuses more directly on optimising performance, recovery, body composition, and promoting adaptation to training (28). Accordingly, nut and seed press cakes may represent an underexplored resource to complement traditional approaches. The fibre content of the nuts and seeds that generate these press cakes may further contribute to satiety and gastrointestinal health (29), which are increasingly recognised as relevant to performance (30). Additionally, the low-fat content of some defatted press cakes makes them a candidate for developing lower-calorie protein sources, suitable for athletes in weight category or aesthetic sports.

Incorporating press cake derived products into the diet aligns with some of the goals set out by the EAT-Lancet report (31) regarding sustainability, upcycling and development of affordable sports nutrition products such as those promoted by the International Olympic Committee (32), as they are generated in large quantities and are often underutilised. This pairs with the growing interest in plant-based diets with environmentally conscious consumption, where athletes and consumers are increasingly seeking foods that address nutrient needs and align with sustainable food systems (33, 34). From a nutrient composition perspective, press cakes likely offer scope to enhance the nutritional value of sports foods. However, press cakes can vary in composition depending on the crop and processing method, presenting possible concerns regarding inconsistent nutrient quantity and quality. Some may contain residual anti-nutritional factors or compounds that impair digestibility, such as phytates, tannins, or trypsin inhibitors. Their sensory properties (taste, texture, solubility) and limited history of use in human sports nutrition products may also restrict consumer acceptance (35). Balancing these benefits and drawbacks will be important in determining the feasibility of including press cakes as sports nutrition ingredients.

Given the increasing interest in plant-based diets, sustainability and environmentally conscious consumption habits, a review synthesising current understandings of nut and seed press cake use, and potential applications in a sporting context is warranted. Accordingly, this work explores the nutritional properties, physiological relevance, and practical formulation considerations of nut and seed press cakes in the context of sports nutrition.

## Methods

### Initial scoping review

A scoping review was conducted to identify studies reporting on the use of nut or seed press cakes in a manner relevant to sports nutrition practice. Search and screening processes were performed in accordance with PRISMA-ScR (Preferred Reporting Items for Systematic reviews and Meta-Analyses extension for Scoping Reviews) guidelines (36, 37), and the completed search terms and checklists are provided in Supplementary File 1. The original search protocol was developed by the authors and pre-registered on the Open Science Framework at the following DOI: https://doi.org/10.17605/OSF.IO/KRWUD. Despite comprehensive searches, screening returned very few relevant examples of the use of nut and seed press cakes in sport nutrition or closely related research. Accordingly, the remainder of this work instead provides a comprehensive overview of the nutrient composition of nuts, seeds and their press cakes with proposed applications to sport nutrition.

### Data and information extraction

All nuts discussed in a recent comprehensive review of upcycling practice of nut byproducts were assessed in the current study (38). Seeds that were most frequently identified in the above-described scoping review, and for which press cake data were accessible, were included in this study. Nutrient composition values (protein, energy, total fat, carbohydrate, fibre, amino acids) were sourced preferentially from the Food Standards Australia New Zealand (FSANZ) database where available (39). Details of the sampling process and reporting are viewable in the FSANZ database for each included food item. It is noted that indices of sample variability are not reported within the database and are therefore absent from this work. Where data was not available via the FSANZ database, values were drawn from peer-reviewed academic sources identified through the scoping review and additional manual searches of PubMed, Scopus and EBSCOHost. For any nut or seed where multiple varieties were reported on, the most widely accessible and commonly reported varieties were included [e.g., Marcona almond as reported by Houmy and colleagues (40)]. All nutrient composition data was extracted from the above sources in August 2025 by a member of the research team and verified by second member of the team.

Where necessary, standardised unit conversions were performed to express all values as grams of a given nutrient per 100 g of edible portion for whole nuts/seeds, or grams per 100 g of dry weight for press cakes. When energy values were not provided in the source material, these were calculated using the Atwater method, applying standard conversion factors for macronutrients (protein = 16.7 kJ/g, fat = 37.7 kJ/g, carbohydrate = 16.7 kJ/g), though known limitations of the Atwater method are acknowledged (41). As an indicator of protein quality and digestibility, Digestible Indispensable Amino Acid Scores (DIAAS) were sought as the preferred index in contemporary nutrition research and practice (42). However, where DIAAS data were unavailable, Protein Digestibility Corrected Amino Acid Scores (PDCAAS) were reported instead.

### Development of summary tables and figures

In addition to a nutrient composition summary (Table 1), further tools were developed to clarify the compositional and applied relevance of nut and seed press cakes in the context of sports nutrition. Table 2 summarises published uses of nut and seed press cakes in food products, and examples of commercially available products that use press cakes from included nuts or seeds. Information for Table 2, and in the results text regarding anti-nutritional factors (e.g., phytates, tannins, or trypsin inhibitors) was drawn from the scoping review and supplementary manual searches described above, focusing on studies that examined either compositional characteristics or the incorporation of press cakes into food or beverage products. To identify commercial uses of press cakes in food and beverage products, we conducted targeted web searches using the same terms as in Supplementary File 1, adding “product” and “manufacturer.” Where possible, manufacturer webpages were recorded instead of third-party sites, with an emphasis on Australian suppliers due to the authors’ location.

**Table 1:** Nutrient composition of commonly available nuts, seeds and their press cakes.

Nut/seed	Protein (g/100 g)	% difference W vs P	Energy (kJ/100 g)	Total fat (g/100 g)	Carbohydrate (g/100 g)	Fibre (g/100 g)	Source
Almond	19.7 (35.8)	81.9	2,385 (1592)	50.5 (10.0)	5.4 (36.7)	10.9 (2.4)	W, WA: (39), P: (94), PA: (40)
Brazil	14.4 (43.5)	202.4	2,886 (1692)	68.5 (9.7)	2.4 (35.8)	8.5^#^	W: (39), P, PA: (95), WA: (48)
Cashew	17.0 (34.0)	100.0	2,540 (1166^*^)	49.2 (1.6)	22.9 (32.2)	5.9 (6.2)	W, WA: (39), P: (96)
Hazelnut	14.8 (42.4)	186.8	2,689 (1833^*^)	61.4 (18.9)	5.1 (24.7)	10.4 (16.5)	W: (39), P: (97), PA: (98)
Macadamia	9.2 (25.5)	177.2	3,018 (5581)	74.0^#^	4.5 (35.9)	6.4 (25.2)	W: (39), P, PA: (99)
Peanut	24.7 (58.3)	136.0	2,376 (1613)	47.1 (5.47)	8.9 (25.8)	8.2^#^	W, WA: (39), P: (100), PA: (101)
Pecan	9.8 (21.9)	123.2	2,973 (1669)	71.9 (16.6)	4.9 (40.5)	8.4 (13.0)	W: (39), P: (102)
Pistachio	19.7 (44.0)	123.4	2,542 (1692^*^)	50.6 (18.3)	15.8 (16)	9.0 (12.0)	W: (39), P: (103), WA: (46)
Walnut	14.4 (24.6)	70.8	2,904 (2638^*^)	69.2 (55.7)	3.0 (7.6)	6.4 (9.4)	W: (39), P: (104)
Chia	23.8 (26.7)	12.0	1825 (1173)	29.8 (6.7)	3.1 (5.1)	33.2 (48.1)	W: (39), P, WA, PA: (105)
Flax	21.6 (36.1)	66.9	1883^#^	32.6 (6.5)	2.8^#^	32.9 (34.9)	W: (39), P, PA: (98)
Hemp	24.8 (26.5)	6.8	2,200 (1700)	^#	27.6 (42.6)	27.6 (42.6)	W, P: (106), WA, PA: (98)
Pumpkin	30.2 (50.2)	66.2	2,249 (2100^*^)	45.0 (31.3)	2.1 (4.9)	4.6 (24.5)	W: (39), P: (98, 107) for fibre, PA: (98), WA: (108)
Sesame	22.4 (40.9)	82.5	^#	41.2 (4.0)	^#	3.4 (7.8)	W, P, PA: (109)
Sunflower	24.7 (21.6)	−12.6	2,362 (1504^*^)	48.3 (14.2)	3.1 (36.5)	13.0 (12.6)	W: (39), P, PA: (110)
Watermelon	17.1 (34.7)	103.1	1,540 (1548^*^)	26.5 (0.9)	15.3 (56.0)	39.1^#^	W: (111), P, PA: (112)

Press cake data are presented in parentheses where available.

Sources are presented as W, whole nut/seed; P, press cake; WA, whole nut/seed amino acids, PA, press cake amino acids. ^#^Indicates insufficient information is currently available regarding the nutrient composition of the associated press cake. ^Indicates insufficient information is currently available regarding the amino acid data of the associated whole nut/seed. *Indicates an energy value calculated according to the Atwater method using other nutrient values from that nut, seed or press cake. % Difference W vs P indicates the percentage difference in protein content between a whole nut/seed (W) and its associated press cake (P), where a positive integer indicates greater protein content for P. Where sources reported a range of values, the lower end of that range has been reported here. Sources are the citation(s) where nutrient composition of the whole nut/seed or the associated press cake were obtained, presented as whole nut source (press cake source). Energy (including from dietary fibre), protein, fat, carbohydrate and fibre are reported as grams per 100 g of edible portion of that nut or seed [per Australian Food Composition Database, FSANZ (39)], and as grams per 100 g of dry weight for the associated press cake where accessible. Where values were reported in other units in the original source (e.g., as mg/g, or as mg/g of protein), conversions have been completed. Where sources reported values for multiple varieties of the same nut or seed, the most widely accessible variety has been included here [such as the Marcona variety of almond reported by Houmy and colleagues (40)]. The average of the three Marcona almond harvest years reported by Houmy and colleagues has been reported here. Where available, a defatted press cake has been used [such as the data extracted from Asen and colleagues for peanut press cake (101)]. NB: variability statistics such as standard deviation, range or confidence intervals for nutrient values from sources other than FSANZ are available in the original source documents, as cited in the final column of this table.

**Table 2:** Published and commercial uses of nut and seed press cakes in food products.

Nut/seed	Top producing countries (of whole nut/seed) (113)	Published use in a food product	Commercially available product	Manufacturer/provider
Almond	USA, Spain, Australia	Bread (16)	Low fat almond flour, powdered almond butter	Spice Zen, Macro Mike
Brazil	Brazil, Bolivia, Peru	Food bars (114)	—	—
Cashew	Ivory Coast, India, Vietnam	Extrudates such as pasta or breads (115)	Powdered cashew butter	PB2 Foods
Hazelnut	Turkiye, Italy, USA	Milk replacement in ice cream (116)	Defatted hazelnut flour	Goulburn Valley Hazelnuts, Pepo Farms
Macadamia	South Africa, China, Australia (117)	Various baked products and powders (118)	No commercial product found, proposed commercial macadamia flour	Plenty
Peanut	China, India, Nigeria	Bread and other baked products (119)	Defatted peanut flour, powdered peanut butter	Nutrin, Golden Peanut, Mayvers, Macro Mike
Pecan	—	Pecan nut beverage (120)	Pecan Flour	Pepo Farms
Pistachio	USA, Iran, Turkiye	Bread (16)	—	—
Walnut	China, USA, Iran	Bread, Cake (12, 16)	Walnut flour	Pepo Farms
Chia	—	Gluten-free bread (121)	Not readily available, proposed commercial chia seed flour	Onset Worldwide
Flax	Russia, Kazakhstan, Canada	Various breads (21)	Flaxseed fine meal	Red Tractor
Hemp	Canada, Australia, Chile	Sponge cake (122)	Hemp seed protein powder	Macro/Woolworths
Pumpkin	—	None identified	Pumpkin seed flour, plant protein powder	Pepo Farms, Keep it Cleaner
Sesame	Sudan, India, Myanmar	None identified	Sesame flour	Spice Zen
Sunflower	Russia, Ukraine, Argentina	Proposed use in baked goods only	Sunflour	Pepo Farms
Watermelon	—	Proposed use in foods only	Not readily available. Proposed commercial watermelon seed flour	Onset Worldwide

Data on country production were drawn preferentially from Food and Agriculture Organization of the United Nations (FAO) data sheets (113). Unless cited otherwise, all country data are drawn from FAO data sheets. Where no FAO data was available for a given nut or seed (macadamia, pecan, chia, pumpkin, watermelon), alternative sources were sought. A dash (—) indicates where no data or information could be sourced. Country production data reflect nut or seed harvest quantity, rather than press cake outputs, as no reputable data on press cake production was identified. Linseed us used interchangeable on FAO sites. Published uses of press cakes in a food product are included as proof of concept to illustrate feasibility, not as an assessment of effectiveness or quality of product. Reported commercially available products are illustrative rather than exhaustive, alternative or similar commercially available products are likely to exist. For some nuts (e.g., Brazil, cashew), available meals or flours appeared to be derived from the whole nut rather than a true press cake and were accordingly not included.

**Figure 1:**
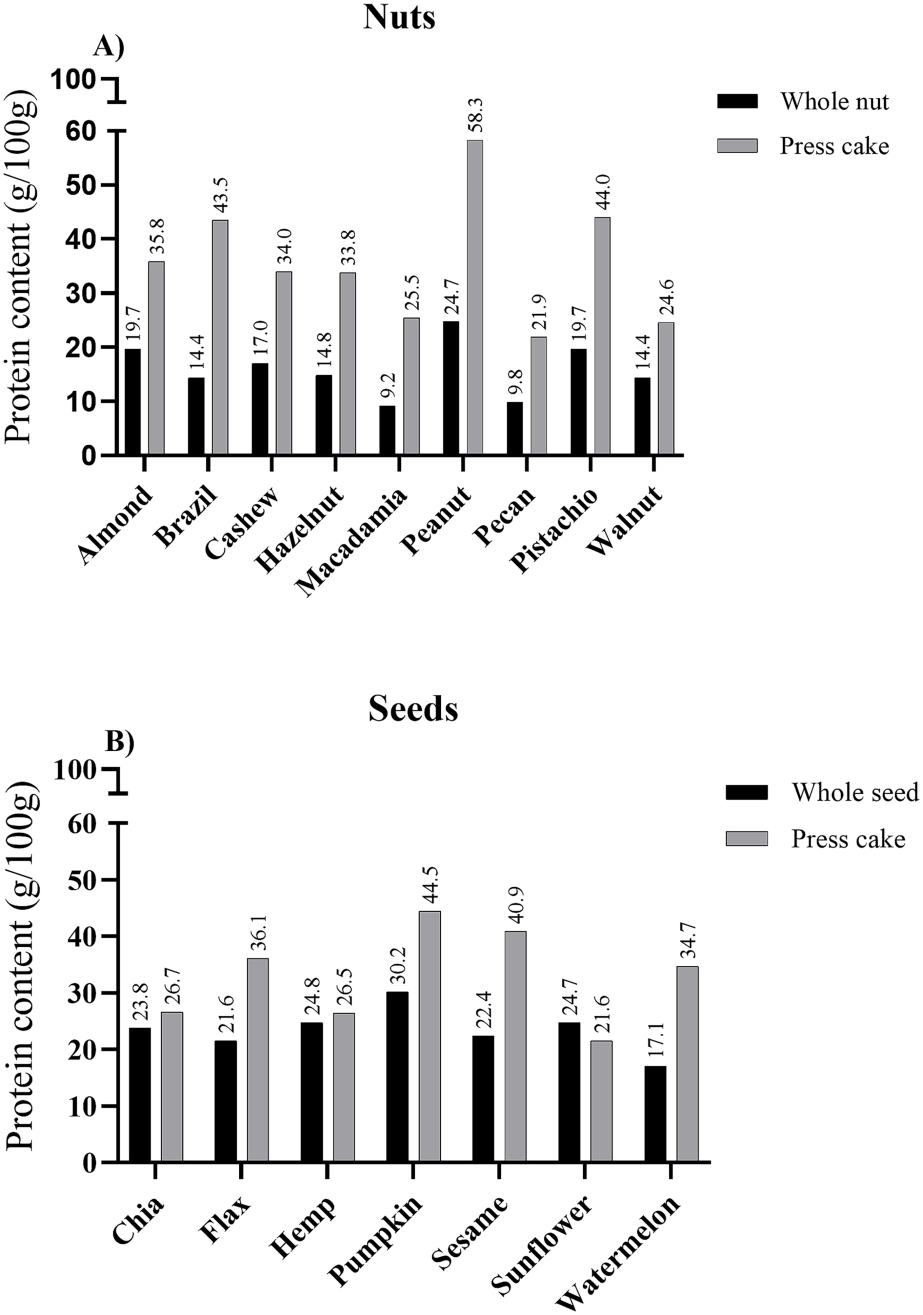
Protein content of various nuts **(A)** and seeds **(B)**, and their press cakes. Black bars represent whole nuts or seeds, grey bars represent their press cake. Sources of the protein content values are reported in Table 1.

**Figure 2:**
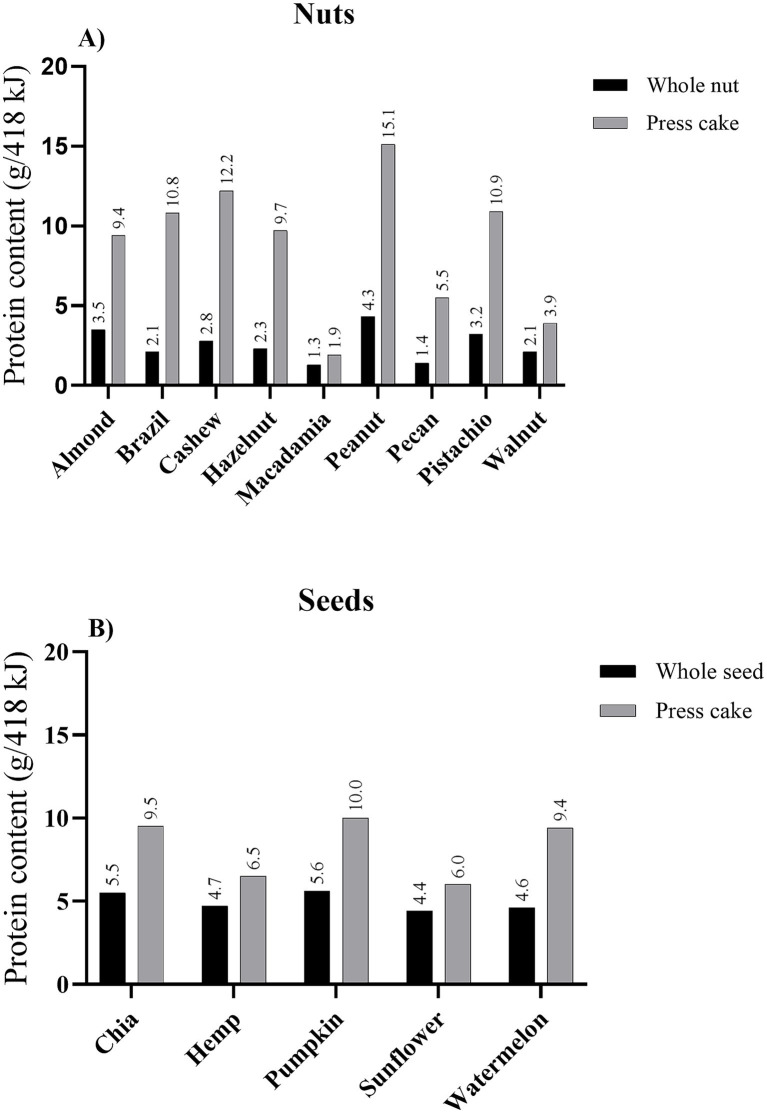
Protein content relative to energy content of various nuts **(A)** and seeds **(B)**, and their press cakes. Black bars represent whole nuts or seeds, grey bars represent their press cake. Sources of the protein content values are reported in Table 1.

**Figure 3:**
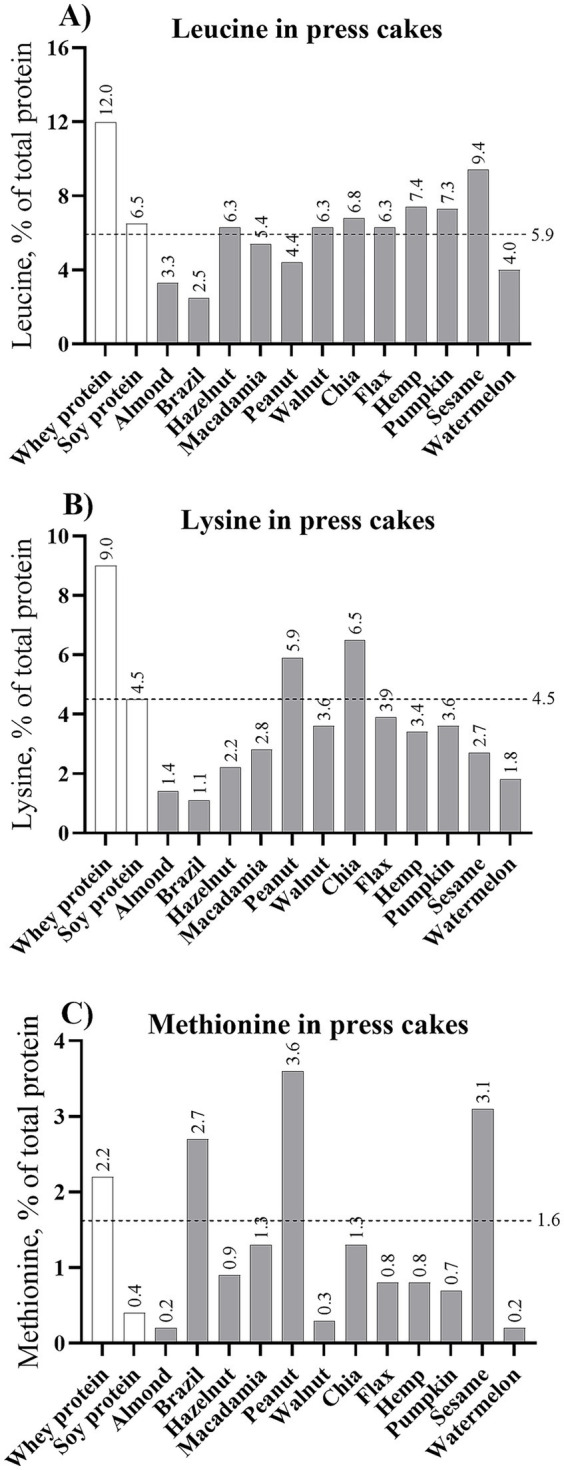
Percentage of protein content that is leucine **(A)**, lysine **(B)** and methionine **(C)** in various nut and seed press cakes, as compared to whey protein and soy protein. Figure is inspired by those reported by Gorissen et al. (43). White bars represent comparison protein powders (whey and soy). Grey bars represent nut or seed press cakes. Dashed line represents the amino acid requirement as reported by WHO in 2007 (44). Only press cakes where amino acid values were accessible have been included in this figure. Sources of amino acid concentrations are reported in Table 1. Where percentage of protein content has been calculated for each amino acid, the protein content of that press cake was drawn from the same source as the amino acid concentration itself (exception of pumpkin seeds where the source that reported amino acid values did not report a usable protein content value, so the protein content reported by FSANZ has been used).

Figure 1 presents the protein content of whole nuts and seeds and their press cakes. In Figure 2, protein content is expressed relative to an energy content of 418 kJ (100 kCal) to standardise comparisons across press cakes with differing energy densities. Figure 3 compares the percentage of protein content comprised of leucine, lysine, and methionine in various nut and seed press cakes with that of whey and soy protein. The figure builds on that presented by Gorissen et al. (43), with the World Health Organisation (WHO) indispensable amino acid requirement profile shown as a dashed line (44). Only press cakes for which amino acid data were available were included to maximise chart clarity. For each amino acid, the percentage contribution to total protein was calculated from values reported in the same source; the exception was pumpkin seed press cake, for which amino acid data were drawn from one source and protein content from FSANZ, due to incomplete reporting.

## Results

The database search identified 725 records, of which 473 remained after duplicate removal. Following title and abstract screening, 12 records were assessed in full, three of which were deemed eligible. Full details of the scoping review search, screening and outcomes can be found in Supplementary File 1. Details of nutrient composition and other characteristics of 16 nuts (*n* = 9) and seeds (*n* = 7) were extracted (Table 1 and Figures 1–3) in subsequent and separate processes from the initial scoping review. FSANZ reported nutrient composition data for all nine nuts, and for chia, flax, pumpkin and sunflower seeds. Press cake data was extracted from 17 academic sources (Tables 1, 2). Amino acid data was extracted from FSANZ for four nuts and seeds, and from 13 other academic sources (Figure 3).

Table 1 summarises compositional values for whole nuts/seeds compared with press cakes. The group mean values for these nutrients are presented here. Relative to whole nuts/seeds, press cakes had higher protein (35.4 vs. 19.3 g/100 g), carbohydrate (27.5 vs. 8.5 g/100 g), and fibre contents (19.9 vs. 14.2 g/100 g), alongside lower energy (1964 vs. 2,425 kJ/100 g) and total fat (14.3 vs. 51.1 g/100 g).

Few DIAAS or PDCAAS values were attainable for any nuts, seeds or press cakes. DIAAS [no limit on values, foods tend to cluster around 100–140 (45)] of 86 for whole pistachios (46), and 35 for peanut press cakes (47) have been reported, with lysine as the first limiting amino acid of both. PDCAAS (highest possible value of 100%) were reported for whole almonds (score = 47.8), Brazil nuts (score = 63.3), cashews (score = 90.3) and peanuts (score = 69.3), each with lysine reported as the limiting amino acid (48, 49).

Across included press cakes, reported anti-nutrients included phytates and polyphenolic compounds, and in some cases cyanogenic glycosides. These mostly tend to be manageable through typical food-processing methods. Phytates, which can reduce absorption efficiency of some minerals (e.g., iron and zinc) (50, 51), are reportedly present in press cakes derived from almonds, peanuts, sesame, and sunflower seeds (52). Phenolic compounds, like tannins and ellagitannins, are found in almond, walnut, chia, sesame and sunflower seed press cakes (52, 53), with potential negative implications for protein digestion alongside their acknowledged antioxidant benefits (54). Protease inhibitors (specifically trypsin inhibitors), are most noted in peanut and sunflower seed press cakes (52, 55), where protein digestibility may be hampered. Cyanogenic glycosides are a more concerning presence in flax press cakes, given they can release toxic hydrogen cyanide (which interferes with cellular respiration and can cause toxicity at high intake levels), though concentrations are reduced to safe levels via heat treatment (56).

## Discussion

The present review collates compositional and applied evidence on the nutrient content, current uses and translational potential of nut and seed press cakes, identifying nutritional feasibility and an absence of current research within sport nutrition contexts. Despite noted uses of press cakes across food and other industrial sectors, our targeted database searches returned no studies directly investigating their use in sports nutrition. The nutrient data and other characteristics presented within the following sections suggest an opportunity to consider press cakes in the sport nutrition context as environmentally sustainable and nutrient-dense ingredients that could support athlete health and performance.

### Nutrient composition and protein quality of nut and seed press cakes

In comparison to whole nuts and seeds, protein content was higher in 15 of 16 press cakes (sunflower seeds exceed the press cake), with seven nut and one seed press cake containing more than double the protein of the whole nut or seed. When expressing protein content as a function of energy (g/418 kJ), press cakes presented more than double the protein density of the whole nut or seed in eight of 14 comparisons (Figure 2), with several nut press cakes exceeding triple that of the whole nut. Energy content was higher in only two press cakes, and total fat content was not higher in any. Where sufficient data was obtainable, carbohydrate content was higher in all press cakes as compared to whole nuts or seeds, and fibre content was higher in 11 of the 13 press cakes from comparable pairs (Table 1, almonds and sunflower seeds exceed their press cakes). Though comparison between nuts/seeds and press cakes is likely to be most appropriate when comparing a given nut or seed with its corresponding press cake, simple group mean values nevertheless highlight clear compositional shifts associated with oil extraction. Relative to whole nuts/seeds, press cakes possessed substantially higher protein, carbohydrate and fibre contents alongside markedly lower total fat and energy density, as expected following lipid removal. These changes align with fundamental work showing that press cakes retain much of the structural carbohydrate and protein matrix while losing most of the extractable fat (57, 58). This nutrient profile, of higher protein and fibre content alongside lower fat and energy, is particularly well aligned with the nutritional aims of many sport nutrition objectives regarding muscle recovery and lean mass maintenance (59, 60).

The amino acid composition of the press cakes assessed here highlighted both their diversity and potential limitations when compared to established sports nutrition products like whey or soy protein powders. While none exceeded whey protein for leucine or lysine content (Figure 3), seven press cakes (hazelnut, walnut, chia, flax, hemp, pumpkin, sesame) exceeded WHO leucine guidelines (44), a key amino acid for muscle protein synthesis, and peanut and chia press cakes both surpassed WHO targets for lysine (44). However, interpretation of these comparisons in a sporting context are limited by the WHO guidelines being applied to the general population only. Brazil nut, peanut and sesame press cakes were notable for their methionine content, as the only press cakes to exceed both whey and soy protein benchmarks. Most included press cakes exceeded soy protein, another plant-based protein option, for methionine content. These findings indicate that several nut and seed press cakes could contribute meaningfully to total amino acid intake in sports nutrition applications, particularly when consumed as complementary blends or in combination with other protein sources.

### Anti-nutrient and digestibility considerations

Anti-nutrients may be a relevant consideration in sports nutrition, given their potential to impede the bioavailability and functionality of key nutrients that may inform decisions regarding the use of press cakes. However, most nut and seed press cakes appear to pose manageable anti-nutritional challenges, rather than substantive safety challenges. The predominant anti-nutritional factors identified in included press cakes were phytates and polyphenolic compounds. These compounds may reduce mineral bioavailability or protein digestibility, though their functional impact is likely moderated by common processing techniques (thermal/heat treatment, fermentation, or protein isolation, which degrade or remove these anti-nutritional factors). Residual concentrations following these processes are therefore likely to vary depending on manufacturing conditions and the degree of refinement.

Most whole nuts and seeds have phytate content values ranging between 0.20 and 9.42 g/100 g dry weight (61). Given phytic acid is the main phosphorous-storage compound in nuts and seeds (62), most press cakes likely retain some phytate after oil-extraction. Nevertheless, concentrations when accessible to consumers primarily represent nutritional limitation rather than safety concern, with prior literature attributing both potentially negative and beneficial physiological outcomes associated with modified phytate intake (50, 61). Similarly, polyphenols and tannins, may reduce protein digestibility or influence palatability (54), but are also associated with antioxidant and anti-inflammatory properties (63), potentially offsetting some limitations in sports nutrition contexts. Flax press cake is a notable outlier, with documented presence of cyanogenic glycosides (linustatin, neolinustatin, linamarin) which may release hydrogen cyanide if inadequately processed (56). Accordingly, appropriate heat treatment and manufacturing controls are essential to minimise residual risk. Hemp press cake is comparatively low in anti-nutritional compounds, though regulatory screening for cannabinoids may be necessary in elite sporting contexts where inadvertent exposure to prohibited substances may incur sanctions from governing anti-doping bodies (64). Overall, with appropriate processing, anti-nutrients appear unlikely to pose functional barriers to the use of nut and seed press cakes in sports nutrition formulations, as any residual anti-nutritional effects may not impair muscle function, recovery or cardiovascular functions (65, 66). However, there is currently a lack of sport-specific literature directly confirming these outcomes, and further investigation would be needed to fully quantify functional effects in athletic populations.

Protein quality or digestibility indicators such as DIAAS and PDCAAS are critical when considering the application of nut and seed press cakes in sports nutrition, as they reflect the availability of amino acids for protein synthesis and subsequent muscle health outcomes that would inform the decision to use press cakes. However, it must be acknowledged that there is limited evidence to confirm the effect of either marker on objective outcomes relating to muscle recovery or sports performance. Accordingly, the following discussion is intended only to facilitate comparisons between nuts, seeds and their press cakes. Compared with animal-derived protein sources such as eggs and whey protein (67), which both consistently return very high DIAAS and PDCAAS indicating efficient absorption (68), plant-derived proteins (such as press cakes) generally display lower values, most often limited by lysine. This suggests that while press cakes could feasibly contribute to protein inputs for muscle function, their ability to support maximal muscle repair or performance may be modest when used as a primary protein source. The very limited existing data reported in the results section of this review highlights the potential use of press cakes in sports nutrition but also points to the need for further direct and athlete-specific research to confirm their efficacy. Indeed, studies with leucine-enriched plant protein isolates indicate that MPS responses can be comparable to whey protein when amino acid composition is optimised. Work with almond protein powder shows nitrogen balance similar to whey at equivalent doses (69), but peanut protein powders may be insufficient to support MPS at doses typically administered (70, 71), emphasising the need for dose–response studies or protein blending strategies to maximise anabolic outcomes from plant derived proteins such as nut/seed press cakes.

### Current uses of press cakes in literature and commercial contexts

Many published instances of nut and seed press cake incorporation into food did so in a baked form. This may reflect the physical compatibility of press cakes (dry and fibrous after oil extraction) with flour-based foods and allows simpler substitution of wheat or other flours, requiring minimal other recipe reformulations (8). Baking can also improve sensory properties by modifying or masking bitter tastes from phenolic compounds found in many press cakes (72), and may reduce anti-nutritional factors such as trypsin inhibitors and phytates (73). There is noted precedent for partial flour replacement using other plant products, such as legume or seed flours to enrich fibre or protein content in baked goods, which may have informed these examples of press cake incorporation (74). Baked products typically have reduced water activity, enhancing shelf life and processing feasibility. In sports nutrition, press cakes have been incorporated into smoothies, protein balls, and other nutrient-dense products, aligning with recommendations from practitioners such as Sports Dietitians Australia (75). Commercially, press cakes are most often presented as flours or powders, either as wheat flour replacements in baked goods (e.g., cakes, brownies) or as ingredients in beverages and snack bars. Together, there is academic and commercial evidence and precedent of press cakes being feasibly incorporated into food, especially as baked goods or in smoothies.

### Global production patterns

Given that press cakes are by-products of nut and seed oil production, their availability is inherently tied to where the raw commodities themselves are grown and processed. Understanding national and regional production patterns, therefore, provides meaningful context for potential supply, scalability and sustainability of press cake use in sport nutrition. Drawing primarily on FAO data (Table 2), global production patterns indicate that most nuts and seeds are cultivated across multiple continents, except for the Brazil nut, for which global supply is geographically restricted almost entirely to South America. Across other nuts and seeds assessed in this review, Asia and Europe are the most frequently represented continents among top-producing nations, with North America, South America, Africa and Australia also represented across multiple nuts and seeds. This broad geographic distribution supports a high degree of scalability and resilience in supply chains for the potential expansion of press cake use in sport nutrition. Specific countries with established nut and seed oil industries (e.g., United States of America for almonds, China for peanuts) are particularly well-positioned to support upcycling of press cakes into plant-based protein ingredients or foods. Alternatively, local production of press cake derived sport nutrition products in these areas could reduce waste, lower transport costs and emissions, and align with global shifts towards circular food systems for environmentally responsible performance nutrition (4).

### Translational potential for sport nutrition

Nut and seed press cakes hold theoretical potential for sport nutrition application based on their relative protein and fibre density, with lower fat and energy content as compared to the whole nut or seed they are derived from (see Table 1 and Figures 1, 2). These properties match those of many foods directed at sports performance or recovery, and support potential incorporation into bars, baked goods and smoothies as part of a food-first approach to meeting athlete nutrient requirements. Existing academic and commercial examples illustrate the feasibility of incorporating press cakes into baked or blended formulations. Many nut and seed press cakes already feature in commercially available forms identified as plant protein powders or flours (Table 2), indicating consumer acceptance and technical feasibility. The efficacy of these examples in a sporting context is not yet established, given a broad lack of human intervention trials that assess sporting or exercise performance following press cake ingestion. Accordingly, the proposed applications here require verification through studies assessing dose–response mechanics, digestibility, gastrointestinal tolerability and sensory preferences.

For athletes adhering to a plant-based diet, press cakes offer a means of diversifying protein and fibre sources, and improving amino acid complementarity when combined with other plant protein sources. Press cakes from hazelnuts, peanuts, walnuts, sesame, chia or pumpkin seeds, for example, contain leucine and/or lysine concentrations that help address amino acid insufficiencies that can be characteristic of plant-based foods and diets (74, 76). Expanding the range of plant-derived protein sources in sport nutrition aligns with current trends toward health and sustainability-conscious dietary patterns among athletes (33, 77). Many nuts and seeds are cultivated globally, enabling regional sourcing and reduced transport emissions, which are relevant for athletes and sporting organisations seeking to minimise environmental and ethical footprints through dietary selections (33). Based on the combined characteristics discussed previously, peanut and sesame seed press cakes appear particularly well suited for incorporation into sports nutrition products. While other nut and seed press cakes also exhibit positive traits for use in sport nutrition, peanut and sesame seed press cakes present especially favourable protein density and amino acid concentrations (see Figures 1, 3 and Table 1), as well as manageable anti-nutrient profiles. Their established use in commercial food products and reliable global supply further support feasibility and scalability.

### Proposed sport nutrition recipes incorporating press cakes

Building on these insights, members of the authorship team with experience in applied sports nutrition modified a set of recipes to demonstrate how peanut and almond press cake products could conceivably be incorporated into commonly consumed foods in sports nutrition. Peanut and almond products were selected as they are most readily available from Australian retailers (e.g., Coles Supermarkets Australia Pty Ltd., sports nutrition stores, online retailers) to maximise practical relevance of this exercise. For each recipe, selected ingredients were replaced with press cake products while all other components were kept the same as from the original recipes, which were sourced from Sports Dietitians Australia sites (75). To illustrate the nutritional implications of these substitutions, Table 3 compares the nutrient composition of each original recipe with the corresponding version incorporating a press cake product. In the case of the sweet potato brownies, almond butter was replaced with an equal quantity of peanut or almond press cake ingredient. For the choc banana smoothie, Milo (malted chocolate powder) was replaced with an equal quantity of peanut or almond press cake ingredient, and full-fat milk was replaced with soy milk to maintain a plant-based approach. Full recipe formulations, including ingredient quantities and full recipe nutrient composition, are provided in Supplementary File 2. Commercially available comparator products for both the brownie and the smoothie have also been included in Table 3.

**Table 3:** Nutrient composition details for sport nutrition recipes, for examples where press cake products have been incorporated, and for comparator products.

Sweet potato brownies				Comparator
Nutrient (per serve, 8 per recipe)	Original recipe	Almond press cake recipe	Peanut press cake recipe	Musashi protein wafer bar (chocolate, 40 g)
Energy (kJ)	1,598	1,290	1,338	851
Protein (g)	2.1	2.2	5.7	11.6
Fat, total (g)	1.8	1.2	2.2	13.2
Carbohydrate (g)	13.0	13.2	15.1	9.1
Sugars (g)	2.3	2.3	3.4	4.1
Fibre (g)	1.6	1.7	2.3	0.0
Sodium (mg)	334	342	394	59
Cost of key ingredient(s) (AUD)	$0.77	$2.82	$1.75	$3.50

Choc banana smoothie				Comparator
Nutrient (per serve, 2 per recipe)	Original recipe	Almond press cake recipe	Peanut press cake recipe	Rokeby protein smoothie (Dutch chocolate, 425 mL)
Energy (kJ)	1,628	1721	1736	1,200
Protein (g)	11.2	10.4	22.9	30.0
Fat, total (g)	10.9	9.1	12.7	6.4
Carbohydrate (g)	50.5	48.2	55.3	27.2
Sugars (g)	24.0	19.6	23.5	25.1
Fibre (g)	5.5	6.4	8.7	-
Sodium (mg)	77	88	275	106
Cost of key ingredient (s) (AUD)	$0.33	$0.93	$0.77	$4.70

Nutrient composition values are reported on a per serve of final recipe basis. “Cost of key ingredient(s) to make full recipe (AUD)” is only the cost of the ingredient(s) that was replaced or the press cake ingredient, to produce the whole recipe, and are reported in Australia Dollars (AUD) as recorded on 28/11/2025. Nutrient composition data for the original recipes are drawn from SDA sites (75). Comparator items are commercially available products that are sold as protein sources, prices are reported per one unit of that product (AUD) as of 9/12/2025.

Across both recipe sets modified with press cake ingredients, nutrient profiles were broadly comparable to the original recipes (Table 3). Peanut press cake substitution markedly increased protein content relative to both the original and almond press cake recipes, with a modest increase in fibre. In contrast, total fat content, energy density and carbohydrate content were largely similar across original and press cake recipes. Notably, the sodium content was higher in peanut press cake recipes, an important consideration for sports nutrition product positioning, as this may be considered advantageous for sports performed under heat stress (78) but less desirable for general health or recovery (79, 80). This likely reflects added salt to improve palatability in commercially available press cake products. Comparator products (protein bar and protein smoothie) were not nutrient-matched to the recipes, to reflect consumer-available composition, but were selected as broadly comparable products. Both provided substantially more protein, while the protein bar also contained higher fat and lower carbohydrate content as compared to the sweet potato brownie recipes.

As these examples are intended as a proof of concept only, substitutions of press cakes for original ingredients were made solely based on nutritional equivalence and ingredient accessibility, without optimisation for sensory attributes. The impact of press cake inclusion on texture, taste, aroma and acceptability was not evaluated, and further formulation work would be needed to refine these recipes for consumer preference, which could come at the expense of some positive nutritional attributes. Cost considerations should also be acknowledged; both press cake modified recipes were approximately two to three times more expensive than the original, though likely still less expensive per serve than the commercially available comparator products.

While these example substitutions demonstrate feasibility from a nutritional standpoint, it is important to consider the functional implications of protein quality for athletic applications. There is evidence that plant-based protein intake, including that derived from press cakes, can support muscle protein synthesis (MPS) when total protein and EAA intake (specifically leucine) are sufficient. For instance, leucine-fortified plant-protein blends have stimulated MPS to a similar extent as whey protein in young adults (81), while reviews highlight that plant-based diets may require a greater total protein intake and focus on amino acid balance to match the anabolic potential of animal proteins in athletes (82). Importantly, the stimulus of resistance training itself remains the primary driver of muscle hypertrophy responses (83), indicating that press cake derived protein could be viable in sports nutrition provided total protein and amino acid needs are still met. These nuances underscore the potential of press cakes as functional ingredients, while further highlighting the need for careful consideration of protein consumption in athletic populations.

### Incorporation in sporting populations

Press cake ingredients could be particularly useful in sports where sustained energy, recovery, and muscle maintenance are critical, and where convenient protein formats are advantageous. For example, resistance-trained athletes or strength sports competitors could benefit from the protein and EAA content of press cakes, supporting MPS and post-exercise recovery (83–85). Endurance athletes, such as long-distance runners or cyclists, may also find value in incorporating press cake products into snacks or smoothies, where the additional protein helps support recovery (3, 86), the lower or similar fat content could reduce energy density, and increased fibre may contribute to gut health (87). Fibre intake pre or during exercise requires careful consideration, however, as it could exacerbate gastrointestinal discomfort if consumed too close to training or competition (88, 89). Beyond nutritional value, the use of press cakes aligns with sustainability goals by upcycling by-products from the food industry, reducing waste, and promoting resource-efficient protein sources (31, 32). In practical terms, products such as protein-enriched brownies or smoothies demonstrate that press cakes can be incorporated into commonly consumed foods that are familiar to athletes, combining convenience, nutrition, and sustainability. Ultimately, further research is needed to quantify the acute and chronic effects of press cake consumption on performance, gut tolerance, and recovery in specific athletic populations.

### Limitations of prior research and current uses

Much of the existing academic literature on nut and seed press cakes has focused on compositional analyses and technological feasibility, rather than human nutrition or performance outcomes. Protein quality indicators such as DIAAS and PDCAAS, which are vital to evaluating suitability for sport nutrition applications, have rarely been determined for press cakes, likely due to complexity, cost and facility requirements of these analyses (90, 91). Accordingly, amino acid profiles are often interpreted without parallel indicators of digestibility or bioavailability, limiting the ability to draw firm conclusions about their functional value for athletes. Additionally, current examples of press cake use in commercial food products are drawn mostly from small-scale or artisanal manufacturers, potentially leading to underrepresentation of true breadth and innovation in this area from other smaller providers who do not maintain a strong online presence.

### Limitations of and comments on the present review

The present review synthesises press cake compositional data, known anti-nutrient characteristics, and documented examples of their use to evaluate translational potential for sport nutrition. However, it does not include direct laboratory or sensory testing of proposed applications. No recipe formulation, stability testing or palatability assessment was conducted, and nutrient data were extracted from multiple sources with varied analytical methods and oil extraction processes. Accordingly, this review can provide only conceptual frameworks for potential sport nutrition applications, without yet validating the functional or sensory feasibility of these products in practice. Commercial examples of nut and seed press cake products are primarily drawn from Australian markets due to author location, so extrapolation to other areas should be considered cautiously.

It is important to emphasise that the discussion presented in this review does not position nut and seed press cakes as dietary supplements or as ergogenic aids, but rather as nutrient-dense ingredients which align with a food-first approach to sport nutrition. They differ from protein isolates/concentrates such as whey, soy, pea and the like in that they have underwent minimal processing following the removal of the oil. Where soy protein isolates/concentrates are made by further processing the soy press cakes, defatted nut and seed powders are less processed and are thus closer in processing terms to being a whole food. Their potential value lies in enhancing the protein and fibre content of commonly consumed foods with a minimally processed product. This aligns with current sport nutrition guidance prioritising whole-food sources of nutrition where possible (92, 93).

### Future directions

Future work may focus on original research evaluating the incorporation of nut and seed press cakes into sport nutrition products and interventions. This could include controlled feeding studies assessing impacts on performance (e.g., strength, endurance time trials, recovery indices) and metabolic responses such as MPS. Comparative studies examining different modes of incorporation, such as baked products versus smoothies or other snacks, may clarify how processing and delivery formats impact digestibility and athlete acceptance. Further research establishing DIAAS or PDCAAS values for a wider selection of press cakes, alongside assessments of amino acid kinetics and postprandial responses in the context of dose response studies, could strengthen the evidence base for the use of press cakes from a mechanistic perspective. Collectively, such work would help determine whether these sustainable by-products can viably contribute to sport nutrition goals within a food-first framework.

## Conclusion

Nut and seed press cakes represent an underutilised, minimally processed by-product of oil production with potential for incorporation into sport nutrition contexts. Their high-protein and fibre content, lower energy and fat content, favourable amino acid profiles, and compatibility with common foods support their possible translational relevance to athletes, particularly those following plant-based or environmentally conscious diets. Future research exploring formulation, digestibility and performance outcomes is necessary to further realise their potential as food-first ingredients for sport nutrition.

With strategic future research, exploring blending press cake powders from a range of nuts and seeds to develop high-protein/fibre foods and drinks, then press cakes could be positioned as a sustainable sports nutrition tool. After all, if whey has been able to make the journey from “Gutter to Gold (2)” then perhaps press cakes can make the journey from the “feed bin to the finish line.”

## Supplementary material

SUPPLEMENTARY FILE 1 Scoping review details.

SUPPLEMENTARY FILE 2 Full recipe composition and nutrient details of example press cake recipe examples.
