# Supplementary material for: Power from the press cake: a scoping and compositional review of defatted nut and seed powders with regards to sports nutrition
Source: Front Nutr. 2026 Jul 8;13:1860103. doi: 10.3389/fnut.2026.1860103 (PMC13388452; doi:10.3389/fnut.2026.1860103)
Supplement: SUPPLEMENTARY FILE 1 — Scoping review details. [file Supplementary_file_1.DOCX]

**Appendix A.** Scoping Review Details

A scoping review was conducted following PRISMA-ScR guidelines. Literature was identified through searches of PubMed, Scopus, and Web of Science from database inception to June 2025. Included sources discussed the nutritional composition, functional application, health outcomes, or sustainability impacts of nut or seed flours relevant to athletic populations. Reference lists of key papers were also screened to identify additional sources

*Eligibility*

Inclusion Criteria:

- English-language publications
- Peer-reviewed articles, reviews, or grey literature such as government reports
- Published since the year 2000
- Studies or articles discussing:
  - Nutritional composition
  - Sports-specific applications
  - Protein quality
  - Food matrix or anti-nutrients
  - Female physiology
  - Product formulation relevant to athletes

Exclusion Criteria:

- Articles focusing on nut/seed oils only
- Non-nutritive uses (e.g., cosmetics)
- Animal-only studies not translatable to human sport settings

*Summary*

Original research published in peer-reviewed journals, doctoral theses, case studies, conference presentations, abstracts and clinical trial reports written in English were included in this scoping review. Letters to the Editor and Review papers were not analysed. The factors outlined in the Participants, Intervention, Comparator, Outcomes and Study Design (PICOS) framework were used to define studies that were eligible for this review, as follows. Participants – Human; Intervention – any nut or seed derived press cake; Comparator – study included placebo or control condition, or a direct comparison to baseline or pre-intervention state; Outcomes – measurements with relevance to sporting or athletic performance, such as aerobic capacity, muscular strength or power, recovery indices; Study Design – intervention or observation studies.

*Search terms, procedures and screening methodology*

Searches were conducted in three databases (PubMed, Scopus and EBSCOHost) from 1^st^ January 2000 to 7^th^ August 2025. Search results were exported to a web-based collaboration software platform, designed for undertaking literature reviews (Covidence, Veritas Health Innovation Ltd, Melbourne, Australia). Duplicates were removed automatically by Covidence and were manually verified by CU. Manual searching of the reference list of each included study was also performed to identify additional eligible studies.

Two reviewers screened the title and abstract of all sources identified in database searches (CU, VT). Full-text articles were then screened by both reviewers for confirmation of inclusion. If full text was not available, the authors were contacted to obtain access. Cause of exclusion at the full-text stage was recorded and is presented in the PRISM-ScR flow diagram in Figure 1. Where disagreement occurred at either stage, a third reviewer was consulted (DLH).

*Summary of search term categories*

Searches all included the following keywords (with format modifications according to database requirements):

| **Group of terms** | **Joining** | **Specific terms** |
| --- | --- | --- |
| Product | AND | Nut, seed, nut flour, seed flour, presscake, press cake, oilseed, oil seed, oilseed cake, oil seed cake, defatted nut, defatted seed, seed powder, nut protein powder, seed protein powder, butter powder, nut butter powder, seed butter powder, seed butter, nut powder |
| Nuts/seeds | AND | Almond, brazil nut, cashew, hazelnut, macadamia, peanut, pecan, pine nut, pistachio, walnut, pumpkin seed, sunflower seed, chia, flax, hemp, sesame, quinoa, poppy, watermelon seed |
| Performance/nutrition | AND | Sports nutrition, sport nutrition, dietary supplement, diet, athlete, exercise, sport, training, performance, endurance, ergogenic, strength, recovery, body composition, muscle, competition, fatigue, power, resistance training, elite |
| Other relevant uses | OR | Protein quality, amino acid profile, digestibility, bioavailability, antinutrient, sustainable, sustainability, upcycle |
| Other not relevant uses | NOT | Cosmetic, dermatology, skin cream, biodesel, lubricant, animal feed, livestock, poultry, agriculture, soy, isolate |

**PUBMED**

**Search terms –** ( "nut flour*"[tiab] OR "seed flour*"[tiab] OR "nut meal*"[tiab] OR "seed meal*"[tiab] OR presscake*[tiab] OR "press cake*"[tiab] OR "oilseed meal*"[tiab] OR "oilseed cake*"[tiab] OR "defatted nut"[tiab] OR "defatted seed"[tiab] OR "protein flour*"[tiab] OR "protein meal*"[tiab] OR "seed powder*"[tiab] OR "nut protein powder*"[tiab] OR "seed protein powder*"[tiab] OR "butter powder*"[tiab] OR "nut butter powder*"[tiab] OR "seed butter powder*"[tiab] OR "nut powder*"[tiab] OR "seed butter*"[tiab] OR ( ( almond*[tiab] OR "brazil nut*"[tiab] OR cashew*[tiab] OR hazelnut*[tiab] OR macadamia[tiab] OR peanut*[tiab] OR pecan*[tiab] OR "pine nut*"[tiab] OR pistachio*[tiab] OR walnut*[tiab] OR "pumpkin seed*"[tiab] OR "sunflower seed*"[tiab] OR chia[tiab] OR flax[tiab] OR hemp[tiab] OR sesame[tiab] OR quinoa[tiab] OR poppy[tiab] OR "watermelon seed*"[tiab] ) AND ( "protein supplement*"[tiab] OR "protein powder*"[tiab] OR "dietary protein"[tiab] OR "protein ingestion"[tiab] OR "protein intake"[tiab] OR "dietary supplement*"[tiab] ) ) ) AND ( exercise[tiab] OR "exercise trial"[tiab] OR "resistance training"[tiab] OR endurance[tiab] OR "endurance training"[tiab] OR performance[tiab] OR ergogenic[tiab] OR strength[tiab] OR recovery[tiab] OR "body composition"[tiab] OR muscle[tiab] OR hypertrophy[tiab] OR fatigue[tiab] OR power[tiab] ) AND "humans"[MeSH Terms] NOT ( cosmetic*[tiab] OR dermatology[tiab] OR "skin cream"[tiab] OR biodiesel[tiab] OR lubricant*[tiab] OR "animal feed"[tiab] OR livestock[tiab] OR poultry[tiab] OR broiler*[tiab] OR chicken*[tiab] OR pig*[tiab] OR swine[tiab] OR cattle[tiab] OR cow*[tiab] OR beef[tiab] OR sheep[tiab] OR lamb*[tiab] OR goat*[tiab] OR aquaculture[tiab] OR fish[tiab] OR trout[tiab] OR tilapia[tiab] OR carp[tiab] OR salmon[tiab] OR rat*[tiab] OR mice[tiab] OR mouse[tiab] OR agriculture[tiab] OR sensory[tiab] OR palatability[tiab] OR flavor[tiab] OR taste[tiab] OR soy[tiab] OR isolate[tiab] OR "soy isolate*"[tiab] )

**Number of hits –** 141 (one duplicate within, 140 final)

**SCOPUS**

**Search terms –** TITLE-ABS-KEY ( "nut flour*" OR "seed flour*" OR "nut meal*" OR "seed meal*" OR presscake* OR "press cake*" OR "oilseed meal*" OR "oilseed cake*" OR "defatted nut" OR "defatted seed" OR "protein flour*" OR "protein meal*" OR "seed powder*" OR "nut protein powder*" OR "seed protein powder*" OR "butter powder*" OR "nut butter powder*" OR "seed butter powder*" OR "nut powder*" OR "seed butter*" OR ( ( almond* OR "brazil nut*" OR cashew* OR hazelnut* OR macadamia OR peanut* OR pecan* OR "pine nut*" OR pistachio* OR walnut* OR "pumpkin seed*" OR "sunflower seed*" OR chia OR flax OR hemp OR sesame OR quinoa OR poppy OR "watermelon seed*" ) AND ( "protein supplement*" OR "protein powder*" OR "dietary protein" OR "protein ingestion" OR "protein intake" OR "dietary supplement*" ) ) ) AND TITLE-ABS-KEY ( exercise OR "exercise trial" OR "resistance training" OR endurance OR "endurance training" OR performance OR ergogenic OR strength OR recovery OR "body composition" OR muscle OR hypertrophy OR fatigue OR power ) AND TITLE-ABS-KEY ( human* ) NOT TITLE-ABS-KEY ( cosmetic* OR dermatology OR biodiesel OR lubricant* OR "animal feed" OR livestock OR poultry OR broiler* OR chicken* OR pig* OR swine OR cattle OR cow* OR beef OR sheep OR lamb* OR goat* OR aquaculture OR fish OR trout OR tilapia OR carp OR salmon OR rat* OR mice OR mouse OR agriculture OR sensory OR palatability OR flavor OR taste OR soy OR isolate OR "soy isolate*" )

**Number of hits –** 211

**EBSCOHost**

**Search terms –** ( "nut flour*" OR "seed flour*" OR "nut meal*" OR "seed meal*" OR presscake* OR "press cake*" OR "oilseed meal*" OR "oilseed cake*" OR "defatted nut" OR "defatted seed" OR "protein flour*" OR "protein meal*" OR "seed powder*" OR "nut protein powder*" OR "seed protein powder*" OR "butter powder*" OR "nut butter powder*" OR "seed butter powder*" OR "nut powder*" OR "seed butter*" OR ( ( almond* OR "brazil nut*" OR cashew* OR hazelnut* OR macadamia OR peanut* OR pecan* OR "pine nut*" OR pistachio* OR walnut* OR "pumpkin seed*" OR "sunflower seed*" OR chia OR flax OR hemp OR sesame OR quinoa OR poppy OR "watermelon seed*" ) AND ( "protein supplement*" OR "protein powder*" OR "dietary protein" OR "protein ingestion" OR "protein intake" OR "dietary supplement*" ) ) )) AND ( exercise OR "exercise trial" OR "resistance training" OR endurance OR "endurance training" OR performance OR ergogenic OR strength OR recovery OR "body composition" OR muscle OR hypertrophy OR fatigue OR power )) AND ( human* )) NOT ( cosmetic* OR dermatology OR "skin cream" OR biodiesel OR lubricant* OR "animal feed" OR livestock OR poultry OR broiler* OR chicken* OR pig* OR swine OR cattle OR cow* OR beef OR sheep OR lamb* OR goat* OR aquaculture OR fish OR trout OR tilapia OR carp OR salmon OR rat* OR mice OR mouse OR agriculture OR sensory OR palatability OR flavor OR taste OR soy OR isolate OR "soy isolate*" ))

**Number of hits –** 373

**Total duplicates removed by Covidence on upload –** 251 (VERIFIED BY CU)

**Total unique results from P, S and EH –** 473

*PRISMA-ScR diagram, exported directly from Covidence*

Studies from databases/registers **(n = 725)**

EBSCOHost (n = 373)

Scopus (n = 211)

PubMed (n = 141)

References from other sources **(n = )**

Citation searching (n = )

Grey literature (n = )

**Identification**

Included studies ongoing **(n = 0)**

Studies awaiting classification **(n = 0)**

Studies included in review **(n = 3)**

Studies excluded **(n = 461)**

Studies not retrieved **(n = 0)**

Studies assessed for eligibility **(n = 12)**

Studies sought for retrieval **(n = 12)**

Studies screened **(n = 473)**

Studies excluded **(n = 9)**

Wrong Intervention (n = 6)

Wrong Outcome (n = 2)

Wrong Patient Population (n = 1)

References removed **(n = 252)**

Duplicates identified manually (n = 1)

Duplicates identified by Covidence (n = 251)

Marked as ineligible by automation tools (n = 0)

Other reasons (n = )

**Screening**

**Included**
