# Supplementary material for: Power from the press cake: a scoping and compositional review of defatted nut and seed powders with regards to sports nutrition
Source: Front Nutr. 2026 Jul 8;13:1860103. doi: 10.3389/fnut.2026.1860103 (PMC13388452; doi:10.3389/fnut.2026.1860103)
Supplement: SUPPLEMENTARY FILE 2 — Full recipe composition and nutrient details of example press cake recipe examples. [file Supplementary_file_2.DOCX]

*Table 4 –* Nutrient composition details for WHOLE sport nutrition recipes, for examples where press cake products have been incorporated, and for comparator products.

| **Sweet potato brownies** | | | | **Comparator** |
| --- | --- | --- | --- | --- |
| **Nutrient (whole recipe)** | **Original recipe** | **Almond press cake recipe** | **Peanut press cake recipe** | **Musashi Protein Wafer Bar (chocolate, 40g)** |
| Energy (kJ) | 12788 | 10318 | 10707 | 851 |
| Protein (g) | 16.7 | 17.9 | 45.4 | 11.6 |
| Fat, total (g) | 14.4 | 9.2 | 17.3 | 13.2 |
| Carbohydrate (g) | 104.1 | 105.6 | 121.1 | 9.1 |
| Sugars (g) | 18.4 | 18.6 | 27.2 | 4.1 |
| Fibre (g) | 13.0 | 13.3 | 18.2 | 0.0 |
| Sodium (mg) | 2669 | 2739 | 3149 | 59 |
| Cost of key ingredient to make full recipe (AUD) | $6.19 | $22.56 | $14.02 | $3.50 |
| **Choc banana smoothie** | | | | **Comparator** |
| **Nutrient (whole recipe)** | **Original recipe** | **Almond press cake recipe** | **Peanut press cake recipe** | **Rokeby Protein Smoothie (Dutch chocolate, 425 mL)** |
| Energy (kJ) | 3256 | 3442 | 3472 | 1200 |
| Protein (g) | 22.5 | 20.7 | 45.9 | 30.0 |
| Fat, total (g) | 21.9 | 18.1 | 25.5 | 6.4 |
| Carbohydrate (g) | 101.0 | 96.4 | 110.6 | 27.2 |
| Sugars (g) | 48.0 | 39.2 | 47.0 | 25.1 |
| Fibre (g) | 11.0 | 12.8 | 17.3 | - |
| Sodium (mg) | 155 | 175 | 549 | 106 |
| Cost of key ingredient to make full recipe (AUD) | $0.66 | $1.86 | $1.53 | $4.70 |

*Table 5 –* Recipe composition of sweet potato brownies (original SDA recipe - [Sweet Potato Brownies - Sports Dietitians Australia (SDA)](https://www.sportsdietitians.com.au/nutrition-kitchen/sweet-potato-brownies/)), and choc banana smoothie (original SDA recipe - [Choc Banana Breakfast Smoothie - Sports Dietitians Australia (SDA)](https://www.sportsdietitians.com.au/recipes/choc-banana-breakfast-smoothie-2/))

| **Sweet potato brownies** | | | |
| --- | --- | --- | --- |
| **Ingredient quantity (g)** | **Original recipe** | **Almond press cake recipe** | **Peanut press cake recipe** |
| Sweet potato puree | 180 | 180 | 180 |
| Almond butter | 258 | - | - |
| PB2 Almond Butter | - | 258 | - |
| PB2 Peanut Butter | - | - | 258 |
| Vanilla extract | 5 | 5 | 5 |
| Oat flour (whole grain) | 47 | 47 | 47 |
| Mini chocolate chips (milk) | 88 | 88 | 88 |
| Brown sugar | 147 | 147 | 147 |
| Cocoa powder | 47 | 47 | 47 |
| Baking soda | 7 | 7 | 7 |
| Salt | 20 | 20 | 20 |
| **Choc banana smoothie** | | | |
| **Ingredient quantity (g)** | **Original recipe** | **Almond press cake recipe** | **Peanut press cake recipe** |
| Blueberries (frozen) | 100 | 100 | 100 |
| Full-fat milk | 250 | - | - |
| Soy milk | - | 250 | 250 |
| Greek yogurt | 250 | 250 | 250 |
| Rolled oats | 20 | 20 | 20 |
| Honey | 7 | 7 | 7 |
| Banana (frozen) | 240 | 240 | 240 |
| Milo | 20 | - | - |
| PB2 Powdered Almond Butter | - | 20 | - |
| PB2 Powdered Peanut Butter | - | - | 20 |
